# Draft Genome Sequence of an Aniline-Degrading Bacterium, *Burkholderia* sp. K24

**DOI:** 10.1128/genomeA.01250-14

**Published:** 2014-12-04

**Authors:** Sang-Yeop Lee, Sung Ho Yun, Chi-Won Choi, Dong-Gi Lee, Jong Soon Choi, Hyung-Yeel Kahng, Seung Il Kim

**Affiliations:** a Korea Basic Science Institute, Daejeon, Republic of Korea; b Department of Environmental Education, Sunchon National University, Suncheon, Republic of Korea

## Abstract

*Burkholderia* sp. K24 is an aniline-degrading soil bacterium that utilizes aniline and its analogues as sole carbon and nitrogen sources. Here, we report the draft genome sequence of this strain that consists of 8,344,181 bp, with a G+C content of 61.7%.

## GENOME ANNOUNCEMENT

*Burkholderia* sp. K24, formerly known as *Acinetobacter lwoffii* K24, was screened from an herbicide-contaminated field in Gyeonggi province in Korea (1). The biodegradation activities of *Burkholderia* sp. K24 include various monocyclic aromatic compounds. *Burkholderia* sp. K24 can use aniline and aniline analogues as its sole carbon and energy source. Aniline is predominantly used as a chemical intermediate in dye, agricultural, and rubber industries. Although it is not considered carcinogenic, continuous inhalation of aniline or water contaminated with aniline produces acute or chronic toxic effects. Many aniline-degrading bacteria have large plasmids containing biodegradation gene clusters. *Burkholderia* sp. K24 uses the β-ketoadipate pathway to degrade aromatic compounds (2, 3). Genome data can provide comprehensive information on the biodegradation of many monocyclic or polycyclic aromatic compounds by *Burkholderia* sp. K24 and the metabolic activities of the bacterium. The data can also be used to confirm the identity of metabolic enzymes screened by proteomic studies because previous studies were performed using a public bacterial genome database (4–6).

The draft genome of *Burkholderia* sp. K24 was generated using the Illumina MiSeq platform (San Diego, CA). A paired-end library with an insert size of 250 to 350 bp resulted in 4,701,300 reads, with a read length of 150 bp. From a mate-paired library with an insert size of 5 kb, we obtained 33,144,478 reads, with a read length of 37 bp.

The sequencing data were assembled with CLC Genomics Workbench (CLC bio, Aarhus, Denmark) version 6.0.2 after data quality control using PrinSeq-lite, version 0.20.3 (7). Genome annotation was performed with the RAST (Rapid Annotation using Subsystem Technology) annotation server (8), Integrated Microbial Genomes/Expert Review (IMG/ER, https://img.jgi.doe.gov/cgi-bin/er/main.cgi) (9), and the NCBI Prokaryotic Genomes Annotation Pipeline (PGAP, http://www.ncbi.nlm.nih.gov/genome/annotation_prok/). Sequencing reads of *Burkholderia* sp. K24 were assembled into 33 scaffolds. The total length of the scaffolds was 8,344,181 bp, with a G+C content of 61.7% and an *N*_50_ contig length of 217,840 bp. A total of 7,033 genes were annotated, including 6,904 protein coding genes and 55 RNA genes.

### Nucleotide sequence accession numbers.

This whole-genome shotgun project has been deposited at DDBJ/EMBL/GenBank under the accession no. JMIK00000000. The version described in this paper is version JMIK01000000.
